# Small Ribosomal Protein RPS0 Stimulates Translation Initiation by Mediating 40S-Binding of eIF3 *via* Its Direct Contact with the eIF3a/TIF32 Subunit

**DOI:** 10.1371/journal.pone.0040464

**Published:** 2012-07-05

**Authors:** Tomáš Kouba, István Dányi, Stanislava Gunišová, Vanda Munzarová, Vladislava Vlčková, Lucie Cuchalová, Andreas Neueder, Philipp Milkereit, Leoš Shivaya Valášek

**Affiliations:** 1 Laboratory of Regulation of Gene Expression, Institute of Microbiology ASCR, v.v.i., Prague, The Czech Republic; 2 Institut für Biochemie, Genetik und Mikrobiologie, University of Regensburg, Regensburg, Germany; University of Kent, United Kingdom

## Abstract

The ribosome translates information encoded by mRNAs into proteins in all living cells. In eukaryotes, its small subunit together with a number of eukaryotic initiation factors (eIFs) is responsible for locating the mRNA's translational start to properly decode the genetic message that it carries. This multistep process requires timely and spatially coordinated placement of eIFs on the ribosomal surface. In our long-standing pursuit to map the 40S-binding site of one of the functionally most complex eIFs, yeast multisubunit eIF3, we identified several interactions that placed its major body to the head, beak and shoulder regions of the solvent-exposed side of the 40S subunit. Among them is the interaction between the N-terminal domain (NTD) of the a/TIF32 subunit of eIF3 and the small ribosomal protein RPS0A, residing near the mRNA exit channel. Previously, we demonstrated that the N-terminal truncation of 200 residues in *tif32-Δ8* significantly reduced association of eIF3 and other eIFs with 40S ribosomes *in vivo* and severely impaired translation reinitiation that eIF3 ensures. Here we show that not the first but the next 200 residues of a/TIF32 specifically interact with RPS0A via its extreme C-terminal tail (CTT). Detailed analysis of the RPS0A conditional depletion mutant revealed a marked drop in the polysome to monosome ratio suggesting that the initiation rates of cells grown under non-permissive conditions were significantly impaired. Indeed, amounts of eIF3 and other eIFs associated with 40S subunits in the pre-initiation complexes in the RPS0A-depleted cells were found reduced; consistently, to the similar extent as in the *tif32-Δ8* cells. Similar but less pronounced effects were also observed with the viable CTT-less mutant of RPS0A. Together we conclude that the interaction between the flexible RPS0A-CTT and the residues 200–400 of the a/TIF32-NTD significantly stimulates attachment of eIF3 and its associated eIFs to small ribosomal subunits in vivo.

## Introduction

Initiation of protein synthesis is one of the key points in regulation of gene expression in eukaryotes, playing a role in processes from neuronal function to development. It requires the coordinated action of large cellular ribonucleoprotein assemblies called ribosomes, initiation Met-tRNA_i_^Met^, mRNA, and at least 12 eukaryotic initiation factors (eIFs). Among them, the multiprotein eIF3 complex deserves a special attention owing to a broad range of functions that it is believed to promote (reviewed in [1]). eIF3, together with eIFs 1, 1A and 5 promotes recruitment of the Met-tRNA_i_^Met^·eIF2·GTP ternary complex (TC) to the small 40S ribosomal subunit (40S) to assemble the 43S pre-initiation complex (PIC) [1]. Furthermore, it significantly stimulates mRNA recruitment to the 43S PICs in co-operation with the poly(A)-binding protein and eIF4F complex, containing the mRNA cap-binding factor eIF4E [2]–[5]. At least in yeast, eIF3 was also implicated in promoting the scanning of the mRNA's 5′ untranslated region (UTR) for the nearest AUG start codon to be recognized by the anti-codon of Met-tRNA_i_^Met^ [6], [7]. Finally, recent reports ascribe eIF3 a coordinating role also in the intricate AUG recognition process by itself [8]–[12].

**Figure 1:**
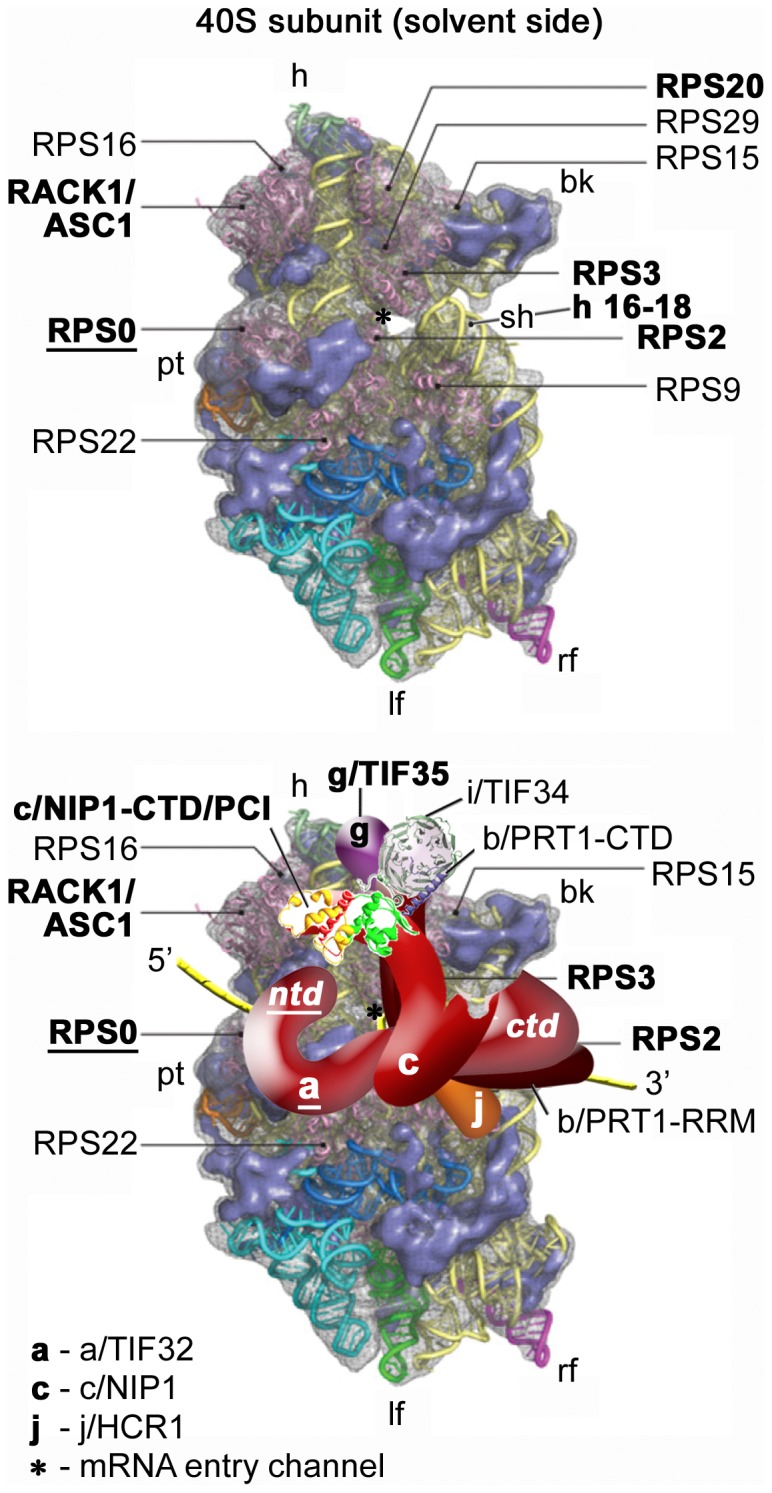
Model of the hypothetical location of eIF3 on the *S. cerevisiae* small ribosomal subunit. (Upper panel) The Cryo-EM reconstruction of the 40S subunit is shown from the solvent side with ribosomal RNA represented as tubes. Ribosomal proteins, with known homologs and placement, are shown as pink cartoons and labeled (adapted from [40]). The positions of helices 16–18 of 18S rRNA, and ribosomal proteins RACK1/ASC1, RPS2, 3, and 20 are highlighted in bold. The position of RPS0, the subject of this study, is highlighted in bold and underlined. The mRNA entry channel is designated by an asterisk. (Lower panel) Hypothetical location of *S. cerevisiae* eIF3 on the back side of the 40S subunit based on the data presented in this study and elsewhere, including the interactions between RPS0 and the NTD of a/TIF32 (in bold and underlined); the c/NIP1-CTD and RACK1/ASC1; RPS2 and j/HCR1; helices 16–18 of 18S rRNA and RPS2 and 3 with the a/TIF32-CTD; and RPS3 and 20 and g/TIF35 (all in bold). The 3D structural model of the c/NIP1-CTD/PCI domain [20] and the X-ray structure of the yeast i/TIF34 – b/PRT1-CTD complex [12] were used to replace the original schematic representations of the same molecules. The yellow lines represent mRNA.

Flawless execution of all these functions depends on proper and stable association of eIF3 with the small ribosomal subunit mediated by number of interactions between its subunits and the ribosomal components. Given the fact that eIF3 associates together with eIF1, TC, and eIF5 in the Multifactor Complex (MFC) [13]–[15], which enhances the efficiency of the PIC assembly process [4], [7]–[9], [16], [17], it is conceivable that timely and spatially coordinated attachment of eIF3 to the 40S subunit also significantly influences binding of the other MFC components. Among the points of contacts between eIF3 and 40S subunit that have been mapped so far are those between (i) the C-terminal domain (CTD) of a/TIF32 and helices 16–18 of 18S rRNA [18] and small ribosomal proteins RPS2 and RPS3 [11], all of which constitute the mRNA entry channel [19]; (ii) the CTD of j/HCR1 and RPS2 and RPS23 [10]; (iii) the g/TIF35 subunit of eIF3 and the 40S beak proteins RPS3 and RPS20 [6]; (iv) the C-terminal PCI domain of c/NIP1 and RACK1/ASC1/RPS33 and probably also 18S rRNA segments of the solvent-exposed head region [20]; and (v) the N-terminal domain (NTD) of a/TIF32 and RPS0A [18], [21]. Based on these findings we have proposed that eIF3 is attached to the rear, solvent-exposed side of the small ribosomal subunit in the area of the head, beak, and shoulder regions (Figure 1) [6], [20]. However, with the exception of the c/NIP1–ASC1 contact, and partly also that between the a/TIF32-NTD and RPS0A, the true physiological importance of most of these interactions remains to be corroborated by *in vivo* approaches.

Here we completed this type of analysis for the a/TIF32-NTD–RPS0A interaction by analyzing the RPS0A contribution to the stable 40S-binding of eIF3. RPS0A is a non-essential protein situated near the mRNA exit channel on the solvent side of the 40S subunit [22], functionally contributing to its cytoplasmic maturation characterized by a final processing of the 20S rRNA precursor to 18S rRNA (the so called cleavage D) [23]. Yeast genome also contains the RPS0B gene whose protein product is about 95% identical to RPS0A. Deletion of both genes is lethal and one can substitute for the other in a higher copy number [24]. In the recently solved crystal structure of 80S ribosome the last 45 C-terminal residues of RPS0 (residues 208–252) are missing [25] suggesting that they are flexible, exposed to the solvent, where they could interact with some ribosome-binding factors. Interestingly, the sequence of the missing C-terminal tail (CTT) is the only region in RPS0 which slightly differs between the A and B isoforms in *S. cerevisiae*. By generating a conditional depletion mutant of RPS0A we demonstrate that its depletion impairs recruitment and/or stable association of eIF3 and other MFC components with 40S subunits resulting in a significant decrease in the translation initiation rates. Importantly, a viable truncation of the RPS0A-CTT, shown to contribute to the RPS0A affinity towards a/TIF32-NTD *in vitro*, produces similar although less pronounced effects. In support, the FLAG-tagged ΔCTT mutant also loses to wt RPS0A in an *in vivo* competition assay for eIFs recruitment. Hence we conclude that the N-terminal residues 200 through 400 a/TIF32 establish an important intermolecular bridge between eIF3 and the small ribosomal subunit via the flexible CTT of RPS0A.

## Results

### Small ribosomal protein RPS0A interacts with the region spanning amino acid residues 200 and 400 of eIF3a/TIF32 *via* its extreme C-terminus

**Figure 2:**
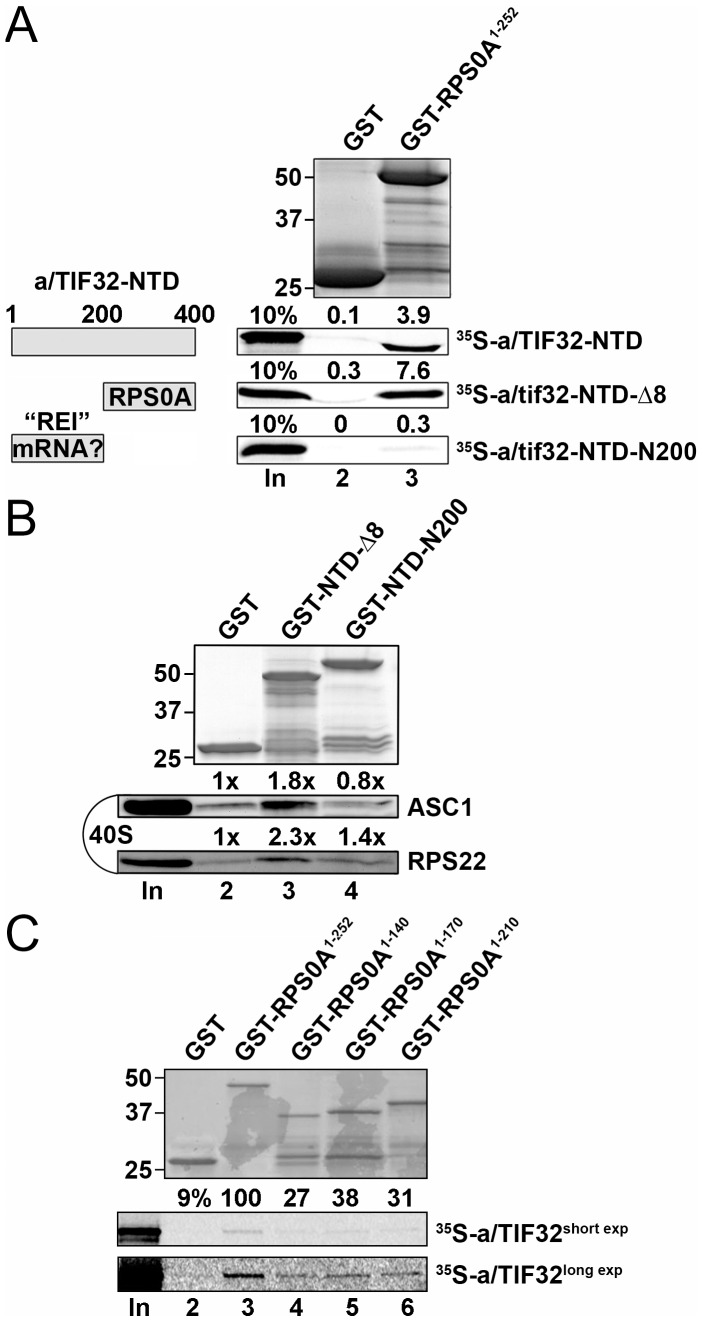
Small ribosomal protein RPS0A interacts with the region spanning amino acid residues 200 and 400 of eIF3a/TIF32 *via* its extreme C-terminal tail *in vitro*. (A) Full-length RPS0A fused to GST (lane 3) and GST alone (lane 2) were tested for binding to ^35^S-labeled wt a/TIF32-NTD [amino acid residues 1–400] and its N- and C-terminal halves; 10% of input amounts added to each reaction is shown in lane 1 (In). The schematic to the right illustrates two discernible regions of the a/TIF32-NTD, one of which promotes reinitiation after translation of short uORFs by contacting specific mRNA regions preceding these uORFs [21], [32], and the other interacts with RPS0A. (B) GST fusions of two consecutive segments of the a/TIF32-NTD in NTD-Δ8 [residues 200–400] and NTD-N2–200 [201–400] in lanes 3 and 4, respectively, or GST alone (lane 2) were tested for binding to the purified wt 40S ribosomal subunits. Lane 1 (In) contains 2.5% of input amounts of 40S subunits added to each reaction mixture. Binding to 40S ribosomes was detected by Western blotting with antibodies against ASC1 and RPS22. (C) Full-length RPS0A (lane 3) and its C-terminal truncations (lanes 4–6) fused to GST, and GST alone (lane 2), were tested for binding to ^35^S-labeled wt a/TIF32; 10% of input amounts added to each reaction is shown in lane 1 (In); short and long exposures are displayed as indicated.

We previously demonstrated that the C-terminal 117 amino acid residues of RPS0A (at positions 135–252) were sufficient for wild type (wt) *in vitro* interaction with the N-terminal half (residues 1–490) of the a/TIF32 subunit of yeast eIF3 [18]. Our yeast two hybrid analysis then suggested that merely the N-terminal 396 residues of a/TIF32 suffice for strong binding to full length RPS0A. Finally, since the deletion of the extreme N-terminal 200 residues of a/TIF32 in the mutant, but still viable *tif32-Δ8* allele markedly reduced 40S-binding affinity of eIF3 *in vivo* [21], we assumed that the RPS0A binding site resides most likely in the first 200 residues of a/TIF32. To test this assumption experimentally, we fused full length RPS0A with the GST moiety and examined the resulting recombinant protein for binding to three N-terminal segments of a/TIF32 synthesized and radiolabeled in rabbit reticulocyte lysates. In accord with our two hybrid data, the longest segment (NTD [1–400]) shows specific binding to GST-RPS0A (Figure 2A, lane 3, second panel). At odds with our prediction, however, removal of the extreme 200 residues (NTD-Δ8 [201–400]) did not abolish but perhaps even improved the quality of this interaction (third panel). This suggests that not the N-terminal stretch of 200 residues but the immediately following one is sufficient for wt binding. Indeed, expression of the 1–200 segment (NTD-N200) completely disrupted binding. Together these data clearly argue that the a/TIF32 residues 201 through 400 are not only sufficient but also necessary for a/TIF32 binding to RPS0A.

To further support this conclusion by demonstrating that the latter a/TIF32 residues contact RPS0A also in the context of the entire ribosome, we decided to examine *in vitro* binding of both halves of the a/TIF32-NTD directly to the purified 40S subunits. To do that, we performed GST pull down assays with the NTD-Δ8 and NTD-N200 fragments fused to GST moiety and the 40S ribosomal subunits isolated from the wt H503 strain. Even though this type of an in vitro assay is not very efficient [20], we repeatedly detected a weak but specific interaction between the minimal RPS0A-binding domain in GST-NTD-Δ8 and purified 40S subunits (Figure 2B, lane 3). In contrast, the extreme N-terminal segment in GST-NTD-N200 showed only background binding comparable to the GST alone control sample (Figure 2B, lanes 4 and 2).

Having shown earlier that the C-terminal 117 residues of RPS0A are sufficient for a/TIF32 binding [18], as noted above, we also wished to examine what part of the RPS0A-CTT is necessary for this contact. Towards this end we generated three C-terminal RPS0A truncations fused to GST and examined their binding affinities towards a/TIF32 *in vitro*. As shown in Figure 2C (lanes 4–6), even the shortest truncation (by 42 residues, lane 6) markedly reduced binding and no further decrease was observed with more progressive truncations. (Note that compared to Figure 2A, we had to use ∼20-fold smaller amounts of GST fusion proteins to reach the ratio at which the decrease in binding was observable; more quantitative measurements will be required in the future to address the effect of individual truncations more specifically.) Nevertheless, it is worth noting that almost exactly this minimal part of RPS0A (precisely the last 45 residues) was found missing in the recently solved crystal structure of the 80S ribosome [25], indicating that it is free to interact with ribosome-accessory factors. Taking together, we propose that the extreme, solvent-exposed RPS0A-CTT is necessary for binding to a/TIF32; at the same time, however, it is not fully sufficient as more N-terminal part of RPS0A is needed for full affinity interaction.

### Rapid depletion of RPS0A significantly decreases translation initiation rates

**Figure 3:**
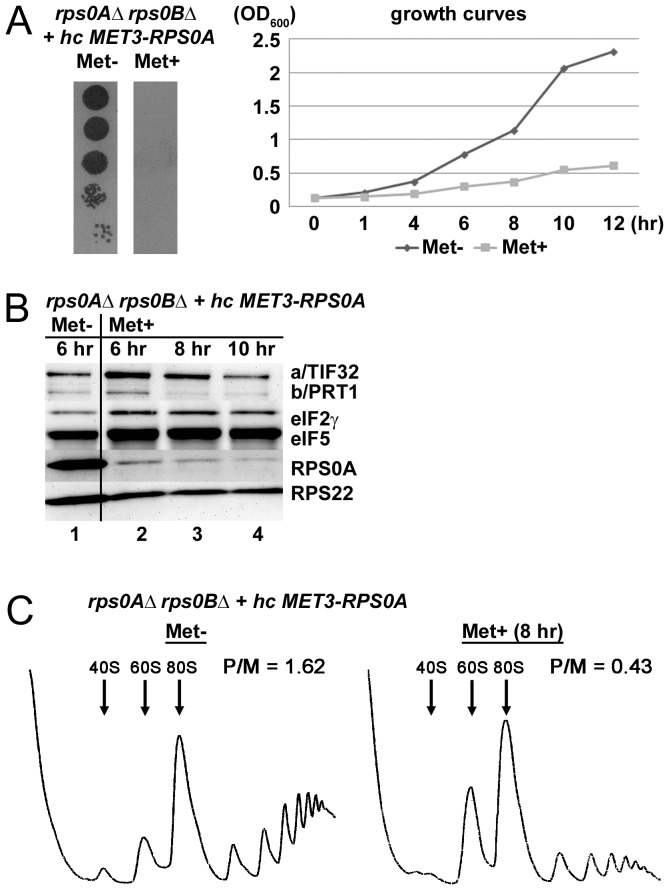
Conditional depletion of RPS0A significantly decreases translation initiation rates. (A) The RPS0A-depletion ceases the growth of mutant cells in non-permissive conditions. Strain YID16 (*rps0aΔ rps0bΔ* YEpMET-RPS0A-U) bearing the *RPPS0A* WT allele under control of *MET3* promoter was spotted in five serial 10-fold dilutions on SD medium +/− methionine and incubated at 30°C for 2.5 days. Growth curves of the same cells grown in liquid SC media lacking methionine at 30°C to an optical density (OD_600_) of 0.15, split into two halves, and further cultivated under the permissive (Met−) and non-permissive (Met+; with 20 mM methionine) conditions for the indicated time intervals at which OD_600_ readings were taken. (B) Rapid depletion of the RPS0A protein in the non-permissive media. The YID16 cells were grown in liquid SC media lacking methionine at 30°C to OD_600_ of 0.3, split into two halves, grown without (lane 1) or with (lanes 2–4) methionine for the indicated time intervals, and WCEs were prepared and subjected to Western analysis using antibodies against the indicated proteins. (C) Rapid depletion of RPS0A dramatically reduces the polysome content. The YID16 cells were cultured under the same conditions as in panel B (the 8 hr interval was chosen for Met^+^ culture) and treated with cycloheximide (5 mg/100 ml) for 5 min prior to harvesting. WCE were prepared and subsequently separated on a 5%–45% sucrose gradient by centrifugation at 39,000 rpm for 2.5 h. The gradients were collected and scanned at 254 nm to visualize the ribosomal species. Positions of 40S, 60S and 80S species are indicated by arrows and polysome to monosome (P/M) ratios are given above the profiles.

As mentioned above, we previously proposed that the RPS0A–a/TIF32 interaction represents an important intermolecular bridge between eIF3 and the small ribosomal subunit [21]. However, the true importance of RPS0A in this bridging interaction has never been directly examined. The yeast genome also contains a *RPS0A* paralogue in *RPS0B* and these two genes can substitute for each other in their essential roles when overexpressed [24]. Hence to address the role of RPS0A in mediating the binding of eIF3 to the 40S, we constructed an *rps0AΔ rps0BΔ* double deletion strain harboring the *RPS0A* allele on a high copy vector under control of the *MET3* promoter. Expression from this promoter can be turned off by supplementing the medium with methionine and we reasoned that a rapid turnover of RPS0A in the cells will allow us to determine the consequences of its depletion on eIF3/MFC association with the 40S ribosome *in vivo*. As expected, addition of methionine to the media promptly ceased the cell growth (Figure 3A). Importantly, this growth defect was fully complemented by co-overexpression of the wt allele under its endogenous promoter from a separate plasmid (Figure S1). To estimate the depletion rates of the RPS0A protein, we grew the *rps0AΔ rps0BΔ* cells in the absence of methionine to an O.D._600_∼0.3, transferred one half of the culture to the media containing methionine, incubated the cells for up to 12 hrs and monitored the RPS0A decrement by Western blotting. As can be seen in Figure 3B, majority of RPS0A was efficiently eliminated after 6–8 hours in the Met^+^ media; however, trace amounts similar to those observed after 8 hr were still detectable even after 10 or 12 hours in non-permissive conditions (lanes 3 and 4, and data not shown). Based on these observations we decided to use the 8-hour interval of depletion as the gold standard for our further analysis. It is worth noting that under these conditions, the growth defect was still reversible on shifting the mutant back to permissive conditions (data not shown).

Having established the depletion conditions, we first analyzed the effect of RPS0A-depletion on the polysome content in *rps0AΔ rps0BΔ* cells cultivated for 8 hrs in the presence of methionine. To compare the amount of translating ribosomes under permissive versus non-permissive conditions, both Met^−^ and Met^+^ cultures were treated with cycloheximide for 5 minutes prior to their harvest to lock the polyribosomes on mRNAs. This so called polysome profile analysis is the most common and established way of monitoring the translational status of cells grown under various conditions [26]. A decrease in the initiation rate results in the typical polysome run-off with a concomitant increase in the amount of free 80S ribosomes, seen as a large monosomal peak in a polysome profile. Indeed, a substantial polysomal run-off reflected in the 3.8-fold reduction in the polysome/monosome (P/M) ratio was found to accompany a loss of RPS0A (Fig. 3C) implicating RPS0A in ensuring optimal initiation rates in wt cells. Even though we cannot completely rule out that some secondary effects arising after 8 hours of depletion could contribute to the observed phenotype, we think that a clear time correlation of the RPS0A-depletion with growth cessation and polysome run-off entitles us to propose that the impact of the RPS0A loss from the cells on translational rates is immediate and hence direct (see also discussion). As expected, we also observed reduced levels of the 40S subunits and, as a consequence, an accumulation of free 60S subunits owing to a defect in 40S biogenesis that occurs in RPS0A or RPS0B mutants [23].

### RPS0A mediates association of eIF3 and its associated eIFs with the 40S ribosomal subunit partly also *via* its C-terminal tail

**Figure 4:**
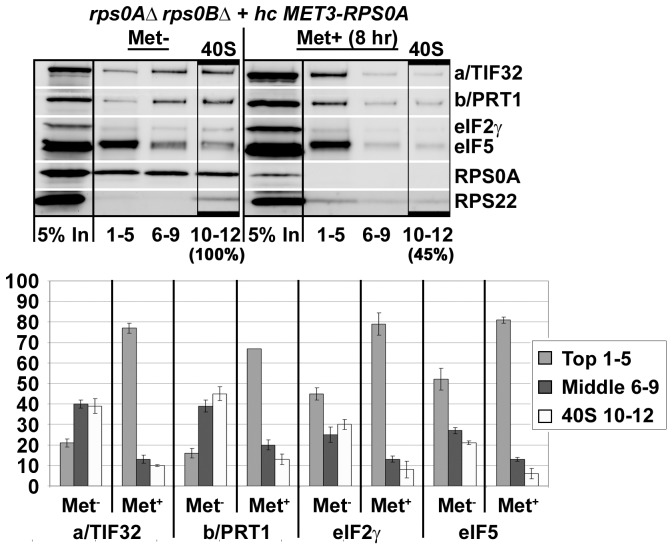
RPS0A mediates *in vivo* association of eIF3 and its associated eIFs with the 40S ribosomal subunit. The same as in Figure 3C, except that the cells were treated with 2% formaldehyde instead of cycloheximide and WCEs were separated on a 7.5%–30% sucrose gradient by centrifugation at 41,000 rpm for 5 h. Proteins from the collected fractions were subjected to Western analysis using antibodies against the proteins listed on the right-hand side of the blots. An aliquot of each WCE was analyzed in parallel (In, input); fractions 1–5, 6–9, and 10–12 were combined. Rectangles indicate fractions where the 43S and 48S pre-initiation complexes sediment (40S); percentages indicate the relative amount of the 40S species in the cells grown under non-permissive versus permissive conditions. Amounts of the each individual factor in the pooled fractions from three independent experiments were quantified by fluorescence imaging, combined and the percentage representation of the signal corresponding to the Top (1–5), Middle (6–9) or 40S (10–12) fractions was calculated and plotted.

To answer the key question of this study, we grew the cells as described above and at the point of harvest, we subjected them to 1 hour treatment with 2% formaldehyde. The advantage of the formaldehyde treatment is that it cross-links eIFs to 40S ribosomes, minimizing dissociation of PICs during sedimentation and thus provides the best available approximation of the native 43S/48S PICs composition *in vivo* [26]. Whole cell extracts (WCEs) derived from thus pre-treated cells were then resolved by velocity sedimentation through 7–30% sucrose gradients, fractioned, and the resulting samples were analyzed for the distribution of selected eIFs and ribosomal proteins across the individual fractions by Western blotting. As shown in Fig. 4, no RPS0A signal was detected in the 40S-containing fractions 10 to 12 derived from the Met^+^ cells that were pooled together. Also note that overexpression of RPS0A under wt Met^−^ conditions produces more protein than it can be incorporated into a natural pool of 40S subunits (Fig. 4, lanes Met^−^, 1–5 and 6–9). Despite an apparent instability of the small ribosomal species (Fig. 4, lanes Met^+^, 1–5 and 6–7), a sizeable fraction of the 40S subunits (∼45% of those found in the Met^−^ cells as calculated from the RPS22 signal using NIH Image J) sedimented in fractions 10–12 indicating that the RPS0A-depleted cells still contain sufficient supply of relatively intact and presumably still active 40S species. (It should be noted that even the immature small ribosomal subunits (deficient in the cleavage D) can interact with eIF3 and other eIFs and fully engage in translation initiation [27]). Importantly, we observed a clear shift in the overall distribution of the amounts of selected eIF3 subunits, and to a similar but perhaps a little less pronounced extent also that of eIF5 and the TC (represented by its eIF2γ subunit), from the 40S-containing fractions 10–12 to the pooled upper fractions 1–5 and 6–9 in the Met^+^ versus Met^−^ cells. With respect to these distribution changes, approximately 4-fold reduction in the amounts of the latter 40S-associated eIFs was detected in fractions 10–12, dropping down from ∼20–45% in Met− to ∼5–10% in Met^+^ cells. These values nicely correlate with those obtained with the *a/tif32-Δ*8 mutant cells lacking the NTD of a/TIF32 [21]. Hence, we conclude that depletion of RPS0A significantly affects 40S-binding of the MFC-associated eIFs, providing a direct evidence for the role of RPS0A in anchoring eIF3 to the solvent side of the small ribosomal subunit.

**Figure 5:**
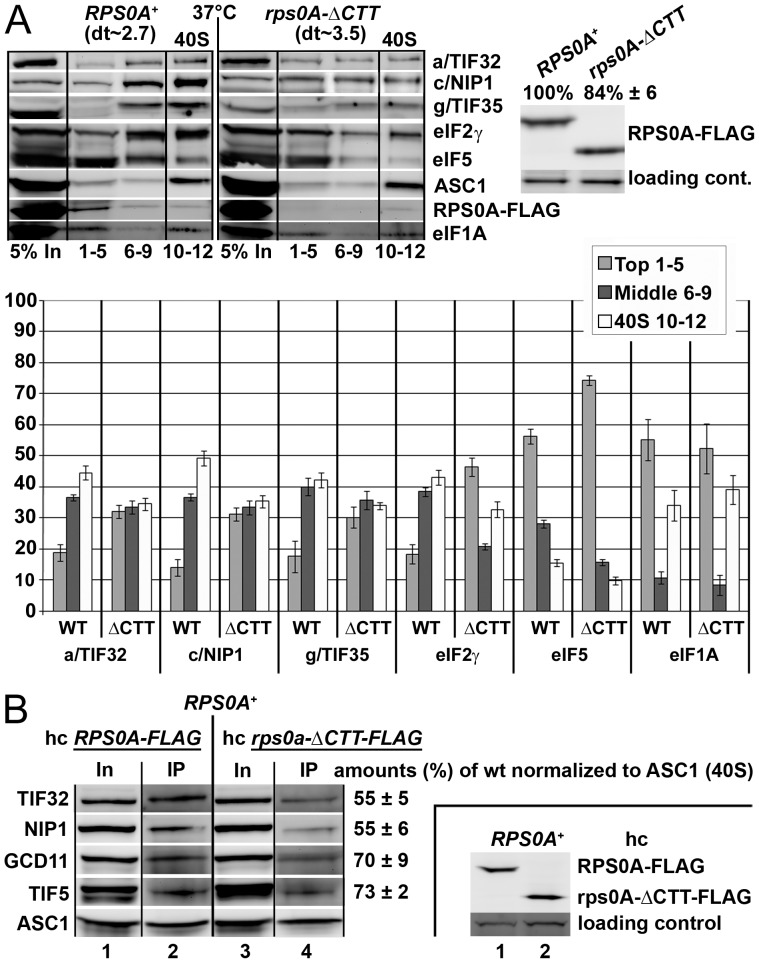
The CTT of RPS0A, making a direct contact with the a/TIF32-NTD, contributes to the RPS0A role in anchoring eIF3 to the small ribosomal subunit. (A) The same as in Figure 4, except that the YID16 cells transformed with a high copy plasmid bearing either wt *RPS0A-FLAG* (in TK156) or *rps0A-ΔCTT-FLAG* (in TK157) alleles under control of the *RPS28* promoter, as the only source of the RPS0A protein product, were grown at 37°C and subsequently subjected to the 2% formaldehyde cross-linking procedure; dt indicates doubling times measured at 37°C. (Expression levels of both FLAG-tagged RPS0A protein variants in TK156 and 157 strains are shown in the right-handed panel). (B) Small ribosomal subunits containing C-terminally truncated RPS0A do not compete well for eIFs recruitment with native ribosomes. A FLAG-tag affinity purification of 40S ribosomes and its associated eIFs from WCEs prepared from the H2880 wt strain overexpressing either wt or C-terminally truncated RPS0A-FLAG followed by Western blotting. (Expression levels of both FLAG-tagged proteins in high copy number in H2880 are shown in the right-handed panel).

Next we wished to demonstrate that the extreme CTT of RPS0A, making a direct contact with the a/TIF32-NTD (Figure 2C), significantly contributes to this role of RPS0A in translation initiation using the *in vivo* formaldehyde cross-linking approach. To do that, we introduced the FLAG-tagged truncation of RPS0A (residues 1–197) – removing all of its flexible C-terminal 45 residues – on high copy plasmid into the YID16 *rps0aΔ rps0bΔ* double deletion strain and analyzed the phenotype of the resulting mutant in comparison with the YID16 strain overexpressing wt RPS0A-FLAG. Importantly, affinity purification of FLAG-tagged full length RPS0A or of this C-terminal truncated form from the corresponding WCEs followed by Northern blotting indicated that both variants were efficiently assembled into 40S subunits and its precursors, and that deletion of the CTT of RPS0A does not lead to major pre-rRNA processing defects ([28] and data not shown). Hence any potential defects associated with this mutant should be directly attributable to the impairment of the RPS0A role in translation only. Growth analysis revealed mild growth phenotype at 37°C (compare doubling times (dt) in Figure 5A) suggesting that the extreme RPS0A-CTT does contribute to the efficiency of the cell proliferation rates. Gradient analysis of the PIC composition then showed modest but reproducible redistribution of eIF3, eIF2 and eIF5 from the 40S-containing fractions into the lighter fractions (Figure 5A). Importantly, the levels of small ribosomal subunits remained unchanged, as expected (compare ASC1 and RPS0A signals in fractions 10–12). The difference in the eIFs amounts in the 40S fractions between the wt and mutant forms of FLAG-tagged RPS0A was between 10–15%. In contrast, the distribution of eIF1A, which is not a part of the MFC, did not change at all. The smaller effect observed in comparison with the depletion of the entire protein is in agreement with i) the fact that besides the CTT, the a/TIF32-NTD contacts also more N-terminal part of RPS0A (Figure 2A); and ii) the modest growth defect associated with the loss of the CTT (Figure 5A). Also note that the wt RPS0A-FLAG but not the CTT-less RPS0A-FLAG protein appears free in the upper fractions suggesting that the wt protein expressed from a high copy plasmid occurs in molar excess over small subunits, as could be expected, and as a result not all RPS0A molecules that are synthesized get incorporated into 40S ribosomes. The fact that no free form of the CTT-less RPS0A-FLAG was observed in the top fractions could be explained by a modestly decreased (by ∼15%) stability of the mutant protein (Figure 5A – left panel) that had, however, no effect on the overall amounts of the 40S subunits, as mentioned above.

Further supporting evidence for the role of the RPS0A-CTT in anchoring eIF3 to the 40S ribosome comes from our last experiment, where we expressed either the FLAG-tagged full length RPS0A or its C-terminally truncated form in otherwise wt cells and affinity purified specifically those ribosomal subunits, which contained either of the FLAG-tagged proteins using anti-FLAG matrix. As shown in Figure 5B, the amounts of 40S-associated eIF3 and eIF2 and eIF5 were reduced by ∼50 and 70%, respectively, when comparing the IP sample containing mutant rps0A-ΔCTT-FLAG versus wt RPS0A-FLAG ribosomes (lanes 2 vs. 4). (Since the rps0A-ΔCTT-FLAG protein runs at the same size as heavy chains of anti-FLAG antibodies (data not shown), obtained results were normalized to the amount of recovered 40S subunits with help of ASC1 instead; comparable expression levels of both FLAG-tagged proteins are shown in Figure 5C.) Together these results demonstrate that 40S ribosomes containing mutant rps0A-ΔCTT-FLAG compete less efficiently with native ribosomes for binding to eIFs than those containing wt RPS0A-FLAG.

## Discussion

Over the last decade we and others have mapped several binding domains within the eIF3 subunits and its associated eIFs that promote stable association of these eIFs with the 40S ribosome [6], [9]–[12], [18], [20], [21], [29], [30]. In many of these cases specific binding partners in ribosomal components were also identified with help of yeast two-hybrid and *in vitro* binding assays; however, a solid *in vivo* evidence underpinning physiological importance of these interactions, especially with respect to ribosomal components, has been largely missing. In fact, the only exception is the recently reported bridging interaction between the PCI domain of the c/NIP1 subunit of eIF3 and the small ribosomal protein RACK1/ASC1, and probably also 18S rRNA segments of the head region [20]. Besides that, N-terminal deletion of RPS5 was previously also shown to affect 40S-binding of eIF3 [31], however, since it is not known whether and how RPS5 contacts eIF3, the molecular nature of this effect is unknown. Here we investigated physiological consequences of disrupting yet another important 40S – eIF3 bridging interaction between RPS0A and a/TIF32 [18], [21] by mutating the RPS0A part of the contact.

Our *in vitro* binding analysis (Figure 2A and C) suggest that the major molecular determinants of this interaction occur in the N-terminal segment of a/TIF32 encompassing the residues 201 through 400 and in the extreme CTT of RPS0A, which is supposed to be free, sticking form the 40S body out into the space [19], [25]. In support, the latter a/TIF32 segment fused to GST but not the preceding one between residues 1 and 200 interacted with the isolated 40S species *in vitro* (Figure 2B). These results seem at odds with our previous finding that the deletion of the first 200 residues in *tif32-Δ8* markedly reduced 40S-binding of eIF3 *in vivo* [21], naturally indicating that the RPS0A binding site resides in this very first 200-residue segment. To reconcile these observations we propose that the entire NTD of a/TIF32 adopts a specific fold that presents the RPS0A-binding site situated between residues 201 through 400 for proper and stable interaction with RPS0A near the mRNA exit pore. Deletion of the first 200 amino acid residues disrupts this fold in such a way that the RPS0A-binding site is, in the context of the entire pre-initiation complex, no longer suitably positioned for a stable binding to RPS0A. It is worth noting that whereas the residues 201 through 400 interact with RPS0A, the extreme N-terminal 200 residues are crucial for efficient reinitiation after translation of short uORFs in yeast by contacting specific mRNA features that precede them (Figure 2A) [21], [32].

An important milestone of our task to implicate RPS0A in promoting assembly of PICs was to demonstrate that mutating *RPS0A* will bear direct consequences in efficiency of translation initiation; i.e. will reduce initiation rates (the P/M ratio) as was observed with our RPS0A-conditional depletion strain (Figure 3). Indeed, it can be argued that depletion of a ribosomal protein, which is an integral part of the export machinery for 20S rRNA-containing pre-40S ribosomes [33], will affect the polysome to monosome ratio primarily by decreasing the levels of mature 40S subunits. However, there are examples of mutant strains (carrying mutations in either 18S rRNA or small ribosomal proteins) with a pronounced defect in 40S biosynthesis showing that a shortage of mature 40S subunits as the primary defect actually leads to a clear decrease not only in the amounts of free 40S subunits and polysomes, but also in the amounts of 80S couples [33], [34]. As a result, these strains display a relatively mild effect on the P/M ratio than could have been expected. This contrasts with our rps0A-depletion data showing significant (∼3.8-fold) reduction in the P/M ratio. Even though it is very likely that a part of this effect can be attributed to a misbalanced ribosome biogenesis, we propose that the main source of the polysome run-off in the rps0A-depletion strain is the inability of the mutant 40S subunits to function properly in translation initiation. An important supporting evidence for this proposal was obtained in our cross-linking experiments, where we showed that the rps0A-depletion impaired association of eIF3, eIF5 and the TC with 40S subunits to a similar extent (∼35%) as observed before with *tif32-Δ8* lacking the a/TIF32-NTD (Figure 4). Further support comes from the analysis of the viable C-terminally truncated *RPS0A-FLAG* allele that, despite having no major defect in ribosome biogenesis (data not shown), also reduced 40S-binding of eIF3 and eIF5 *in vivo*, though to a smaller degree (Figure 5A). Consistently, ribosomes containing rps0A-ΔCTT-FLAG could not compete well with native ribosomes for recruitment of eIFs in otherwise wt cells when compared to the wt “RPS0A-FLAG” ribosomes (Figure 5B). Hence, besides the aforementioned interaction of the c/NIP1-PCI with RACK1/ASC1 and 18S rRNA [20], this work concludes characterization of another important intermolecular bridge spanning between the RPS0A-CTT and the a/TIF32-NTD that anchors the MFC to the small ribosomal subunit in a co-operative manner with another, yet to be identified 40S-eIF3 contacts.

## Materials and Methods

### Yeast strains and plasmids

Strain YID16 (*leu2-3,-112 ura3-52 trp1 rps0a::kanMX3::TRP1 rps0b::kanMX* [YEpMET-RPS0A-U]) was constructed in the following steps. (i) To delete a chromosomal copy of *RPS0A*, two primers, RPS0A-A 5′ AATTAACTTCGTAGGCTGGAACAG 3′ and RPS0A-D 5′ CCAGTTTTGTTCATGGATAAGAGGT 3′, were used to PCR amplify a DNA fragment containing the *rps0A*::*kanMX* allele from strain BY4844 obtained from Research Genetics. The purified DNA fragment was used to transform strain H2880 (*MATa leu2-3,-112 ura3-52 trp1*) [7] to G418 resistance. The deletion of *RPS0A* was verified by PCR. The resulting strain YID08 (*leu2-3,-112 ura3-52 trp1 rps0a::kanMX3*) was transformed with the *kanMX::TRP1* cassette (a *Not*I fragment of M4757 [35]) to tryptophan prototrophy and regained sensitivity to G418, producing yID14 (*leu2-3,-112 ura3-52 trp1 rps0a::kanMX3::TRP1*). (iii) Subsequently, YID14 was introduced with YEpMET-RPS0A-U and a chromosomal copy of *RPS0B* was deleted by transforming the resulting strain by a *rps0b::KanMX4* cassette obtained from BY4741 (Research Genetics) back to G418 resistance using primers BSRPS0A 5′ CATGCATTCATGACAATTTTACCTA 3′ and BSRPS0D 5′ CTAGCATTGAAAACGTATGGTTCTT 3′. Thus generated YID16 was verified by Western analysis using antibodies against RPS0A (see below).

To create TK156 and TK157, YID16 was first transformed with YCpMETRPS0A-W and the Uracil auxotrophy was regained by growing the cells on SD plates containing 5-fluoro-orotic acid. The resulting strain was subsequently transformed with YEp-pRPS28B-FLAG-RPS0A and YEp-pRPS28B-FLAG-RPS0A-ΔCTT *LEU2*-based plasmids, respectively, and the tryptophan auxotrophy was regained by growing the cells in liquid media containing tryptophan; cells that lost YCpMETRPS0A-W were selected based on a growth test on SD plates lacking or supplemented with tryptophan. Thus generated TK156 and TK157 strains were verified by Western blotting analysis using anti-FLAG M2 antibody (Sigma).

H503 (*MAT*a *leu2-3 112 his4-539 trp1 ura3-52 cup1::LEU2/PGK1pG/MFA2pG*) was used for purification of the 40S ribosomal species.

Construction of YEpMET-RPS0A-U was done in the following three steps. (i) A 1222-bp *Bam*HI-*Sal*I fragment from pGBKRPS0A [18] was inserted between *Bam*HI-*Sal*I multicloning sites of YCplac22MET-W (a kind gift of Kim Nasmyth). (ii) Thus created YCpMETRPS0A-W was cut with *Xba*I-*Sph*I and the resulting 1553-bp fragment was inserted between *Xba*I-*Sph*I sites of YEplac112 [36] producing YEpMET-RPS0A-W. (iii) Subsequently, a 2025-bp *Sac*I-*Sph*I fragment from YEpMET-RPS0A-W was inserted between *Sac*I-*Sph*I sites of YEplac195 [36] producing YEpMET-RPS0A-W.

pGEX-RPS0A was constructed in two steps. First, the intron was removed by a fusion PCR using pGBK-RPS0A [18] as a template and the following pair of primers for two separate PCR reactions producing exon 1 (O1-BamHI 5′ AATAGGATCCCATATGTCCTTACCAGCTACTTTTGAC 3′ and BSRPS0exon1-r 5′ TGAAAACATACGGTTCTTGGTGAACTTGAACGTTTCTAGCAC 3′) and exon2 (BSRPS0exon2 5′ CAAGAACCGTATGTTTTCAACGCAAGACC 3′ and O2-KpnI-XhoI-r 5′ TTCTCGAGATGGTACCACTTACCACTCGACGTTGTCAGCATTTTC 3′), respectively. Both PCR products thus obtained were used in a 1∶1 ratio as templates for third PCR amplification using primers O1-BamHI and O2-KpnI-XhoI-r. The resulting PCR product was digested with *Bam*HI and *Xho*I and ligated with *Bam*HI-*Xho*I-cleaved pGEX-4T-1 [37].

pGEX-RPS0A^1–140^, pGEX-RPS0A^1–170^ and pGEX-RPS0A^1–210^ were made by inserting the corresponding *Bam*HI-*Sph*I digested PCR products obtained with primers O1-BamHI and IDRPS0A-1–140-r (5′ ATAGCATGCTTAGTTAACGTAAGAAGCTTCCT 3′) or IDRPS0A-1–170-r (5′ ATAGCATGCATTAAATGGAGTGCTTACCTCTGT 3′) or IDRPS0A-1–210-r (5′ ATATGCATGCATTAAACTTCTTCAGGGTCTCTGT 3′), respectively, using pGEX-RPS0A as a template into *Bam*HI-*Sph*I digested pGEX-RPS0A.

Construction of pT7-TIF32 and pGAD-a/TIF32-NTD was described in [30], [32], respectively. Plasmids pGAD-a/TIF32-NTD-Δ8 and pGAD-a/TIF32-NTD-N200 were generated by inserting the *Nde*I-*Xho*I-digested fragments obtained by PCR using YCp-a/TIF32-His-screen as a template and the following pairs of primers: SGTIF32d8NDEIF 5′ CATCAAGCATATGAGATTAGCTGAAATG 3′ and SGTIF32NTDXHOIR 5′ CCCCCTCGAGTTAATCAAAGTTAACTTCAATG 3′; and SGTIF32NDEIF 5′ GAAGGATCCATATGGCCCCCCCAC 3′ and SGTIF32NTD200XHOIR 5′ CCCCCTCGAGTTACTTGAATTCGTTTTTACGC 3′, respectively, into *Nde*I-*Xho*I-cut pGADT7 (Clontech).

To construct pGEX-a/TIF32-NTD, pGEX-a/TIF32-NTD-Δ8 and pGEX-a/TIF32-NTD-N200, the corresponding a/TIF32-NTD segments were PCR out using the following three sets of primers (VVTIF32NTDXMAIF_5′ CACCCGGGGCATGGCCCCCCCAC 3′ and SGTIF32NTDXHOIR_5′ CCCCCTCGAGTTAATCAAAGTTAACTTCAATG 3′; VVTIF32NTDXMAIF_5′ CACCCGGGGCATGGCCCCCCCAC 3′ and SGTIF32NTDXHOIR_5′ CCCCCTCGAGTTAATCAAAGTTAACTTCAATG 3′; and VVTIF32d8XMAIF_5′ CACCCGGGGCATGAGATTAGCTGAAATGT 3′ and SGTIF32NTDXHOIR_5′ CCCCCTCGAGTTAATCAAAGTTAACTTCAATG 3′) and pT7-a/TIF32-NTD, pT7-a/TIF32-NTD-Δ8 and pT7-a/TIF32-NTD-N200 vectors, respectively, as templates (the reverse primers introduced the *Xma*I restriction site at the 3′ end of all three PCR products). Thus obtained PCR products were cut with *Xho*I and *Xma*I and ligated with *Xho*I*-Xma*I-cleaved pGEX-5X-3 [37].

YEp-pRPS28B-FLAG-RPS0A (request number K421, [28]) is based on vector YEplac195 [36] into which a PCR amplicon, which was produced using oligos O581_5′ TTTTTTGAATTCGCTTATTCATGTTCGAATC 3′ and O582_5′ TTTTTTGGATCCGGTACCCTTATCGTCGTCATCCTTGTAATCCATTGCTGCTC TTTTAGCTTTGC) and yeast genomic DNA as template, and which contains the promoter of *RPS28B* followed by the coding sequence for the FLAG tag, was cloned between the *Eco*RI and *Bam*HI sites producing YEplac195-pRPS28B (request number K349). The *RPS0A* open reading frame lacking the intron was subcloned using *Bam*HI and *Pst*I from YCplac111-pGAL-RPS0A (request number K251, [33]) into K349 to result in vector YEp-pRPS28B-FLAG-RPS0A (request number K421). YEp-pRPS28B-FLAG-RPS0A-ΔCTT (request number K514) was obtained by inserting a PCR amplicon, produced with oligos O490 and O848_5′ TTACTGCAGTTAGATGGACCATGGTTGA 3′ and yeast genomic DNA as template, into the *Bam*HI and *Pst*I sites of K349. Sequencing analysis of K514 revealed a neutral C45T conversion.

### Biochemical methods

GST pull-down assays with GST fusions and *in vitro*-synthesized [^35^S]-labeled polypeptides were conducted as follows. Individual GST-fusion proteins were expressed in *E. coli*, immobilized on glutathione-Sepharose beads and incubated with 10 µl of [^35^S]-labeled potential binding partners at 4°C for 2 h. The beads were washed 3 times with 1 ml of phosphate-buffered saline and bound proteins separated by SDS-PAGE. Gels were first stained with Gelcode Blue Stain Reagent (Pierce) and then subjected to autoradiography. Polysome profile analysis, 2% HCHO cross-linking, WCE preparation and fractionation of extracts for analysis of pre-initiation complexes were carried out as described by [38].

### 40S-binding assay

40S ribosomal subunits were purified as described previously [39]. GST-pull down assays with purified 40S subunits were performed according to [20], [34] with minor modifications. Briefly, the glutathione-Sepharose beads adsorbed with wt GST-a/TIF32-NTD fusion proteins were firstly preincubated with 2 µg/ml total yeast tRNA (Sigma) in the binding buffer B (20 mM HEPES [pH 7.5], 2.5 mM MgCl_2_, 100 mM KCl, 0.1 mM EDTA, and 2 mM DTT) to prevent unspecific binding. After several washing steps with the buffer B, the beads were incubated with purified ribosomal 40S subunits at the final concentration of 0.4 µM in the buffer B supplemented with 0.75% dry milk for 30 min at 22°C. After three washes with the buffer B, the resulting complexes were subjected to SDS-PAGE electrophoresis followed by immunoblotting with anti-ASC1 and anti-RPS22 antibodies.

### FLAG tag co-immunoprecipitation pull-down

FLAG tag co-immunoprecipitation pull-down was performed according to [28] with minor modifications. Transformants of the H2880 wt strain overexpressing either wt or C-terminally truncated RPS0A-FLAG were grown in SD medium at 30°C to an OD_600_ of ∼1, cooled down to 4°C and disrupted by FastPrep-24 (MP Biomedicals) with glass beads in the buffer A200 (200 mM KCl, 20 mM Tris [pH 8], 5 mM Mg(CH_3_C00)_2_, 1 mM DTT, 1 mM PMSF, Complete EDTA-free protease inhibitor cocktail tablet [Roche]). Extracts were clarified by two consecutive centrifugations at 14,000 rpm for 5 and 10 min at 4°C. Triton X-100 was added to the supernatants to 0.2%. The resulting WCEs (300 mg) in a final volume of 250 µl were incubated with 100 µl of anti-FLAG M2 affinity matrix (Sigma) for 60 min at 4°C in A200T (buffer A200, 0.2% Triton X-100). Subsequently, the beads were washed five times with 2 ml of A200 and once with 10 ml of A200T. Co-immunoprecipitated proteins were eluted from the affinity matrix by boiling the samples in the SDS-PAGE loading buffer, loaded on the gel and analyzed by Western blotting.

### Preparation of antibodies against RPS0A

The GST-RPS0A fusion protein encoded by pGEX-RPS0A was expressed in *E. coli* and purified from the WCE by incubation with Glutathione-Sepharose 4B beads (Pharmacia). The isolated protein was resolved by SDS-PAGE (4–20% gels), excised from the gel, and washed with 1× PBS. Rabbits were injected with the purified protein and serum containing polyclonal antibodies against RPS0A was obtained commercially by Apronex (Prague, the Czech Republic).

## Supporting Information

Figure S1 **Growth cessation caused by the RPS0A-depletion under non-permissive conditions is fully reversible by high copy expression of the wt RPS0A allele expressed form its endogenous promoter.** Strain YID16 (*rps0aΔ rps0bΔ* YEpMET-RPS0A-U) bearing the *RPPS0A* WT allele under control of *MET3* promoter was further transformed by YEpRPS0A-L (bearing the wt *RPS0A* allele under control of its own promoter) and the resulting transformant was spotted in four serial 10-fold dilutions on SD medium +/− methionine and incubated at 30°C for 2.5 days.
(PDF)
