# Supplementary material for: Small Ribosomal Protein RPS0 Stimulates Translation Initiation by Mediating 40S-Binding of eIF3 via Its Direct Contact with the eIF3a/TIF32 Subunit
Source: PLoS One. 2012 Jul 5;7(7):e40464. doi: 10.1371/journal.pone.0040464 (PMC3390373; doi:10.1371/journal.pone.0040464)

Figure S1. Growth cessation caused by the RPS0A-depletion under non-permissive conditions is fully reversible by high copy expression of the wt RPS0A allele expressed form its endogenous promoter. Strain YID16 (*rps0aΔ rps0bΔ* YEpMET-RPS0A-U) bearing the *RPPS0A* WT allele under control of *MET3* promoter was further transformed by YEpRPS0A-L (bearing the wt *RPS0A* allele under control of its own promoter) and the resulting transformant was spotted in four serial 10-fold dilutions on SD medium +/- methionine and incubated at 30°C for 2.5 days.

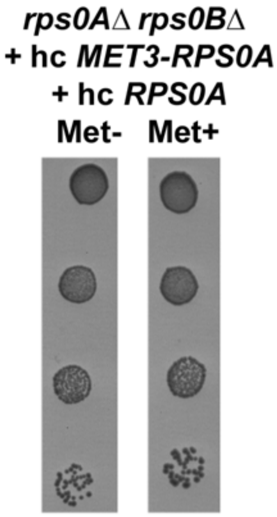

Supplement: Figure S1 — Growth cessation caused by the RPS0A-depletion under non-permissive conditions is fully reversible by high copy expression of the wt RPS0A allele expressed form its endogenous promoter. Strain YID16 (rps0aΔ rps0bΔ YEpMET-RPS0A-U) bearing the RPPS0A WT allele under control of MET3 promoter was further transformed by YEpRPS0A-L (bearing the wt RPS0A allele under control of its own promoter) and the resulting transformant was spotted in four serial 10-fold dilutions on SD medium +/− methionine and incubated at 30°C for 2.5 days. (PDF) [file pone.0040464.s001.pdf]
